# Development of a predictive model for patients with bone metastases referred to palliative radiotherapy: Secondary analysis of a multicenter study (the PRAIS trial)

**DOI:** 10.1002/cam4.70050

**Published:** 2024-10-10

**Authors:** Romina Rossi, Federica Medici, Ragnhild Habberstad, Pal Klepstad, Savino Cilla, Monia Dall'Agata, Stein Kaasa, Augusto Tommaso Caraceni, Alessio Giuseppe Morganti, Marco Maltoni

**Affiliations:** ^1^ Palliative Care Unit IRCCS Istituto Romagnolo per lo Studio dei Tumori (IRST) “Dino Amadori” Meldola Italy; ^2^ Radiation Oncology, Department of Medical and Surgical Sciences (DIMEC) Alma Mater Studiorum University of Bologna Bologna Italy; ^3^ Radiation Oncology IRCCS Azienda Ospedaliero‐Universitaria di Bologna Bologna Italy; ^4^ Department of Clinical and Molecular Medicine Norwegian University of Science and Technology Trondheim Norway; ^5^ Department of Oncology St. Olavs University Hospital Trondheim Norway; ^6^ Department of Circulation and Medical Imaging Norwegian University of Science and Technology Trondheim Norway; ^7^ Department of Anesthesiology and Intensive Care Medicine St Olavs University Hospital Trondheim Norway; ^8^ Medical Physics Unit Responsible Research Hospital Campobasso Italy; ^9^ Unit of Biostatistics and Clinical Trials IRCCS Istituto Romagnolo per lo Studio dei Tumori (IRST) “Dino Amadori” Meldola Italy; ^10^ Department of Oncology Oslo University Hospital Oslo Norway; ^11^ Palliative Care, Pain Therapy and Rehabilitation Unit Fondazione IRCCS Istituto Nazionale dei Tumori Milan Italy; ^12^ Department of Clinical Sciences and Community Health Università degli Studi di Milano Milan Italy; ^13^ Medical Oncology Unit, Department of Medical and Surgical Sciences (DIMEC) Alma Mater Studiorum‐University of Bologna Bologna Italy

**Keywords:** bone metastasis, LASSO, multicenter, palliative therapy, predictive model, radiotherapy

## Abstract

**Background:**

The decision to administer palliative radiotherapy (RT) to patients with bone metastases (BMs), as well as the selection of treatment protocols (dose, fractionation), requires an accurate assessment of survival expectancy. In this study, we aimed to develop three predictive models (PMs) to estimate short‐, intermediate‐, and long‐term overall survival (OS) for patients in this clinical setting.

**Materials and Methods:**

This study constitutes a sub‐analysis of the PRAIS trial, a longitudinal observational study collecting data from patients referred to participating centers to receive palliative RT for cancer‐induced bone pain. Our analysis encompassed 567 patients from the PRAIS trial database. The primary objectives were to ascertain the correlation between clinical and laboratory parameters with the OS rates at three distinct time points (short: 3 weeks; intermediate: 24 weeks; prolonged: 52 weeks) and to construct PMs for prognosis. We employed machine learning techniques, comprising the following steps: (i) identification of reliable prognostic variables and training; (ii) validation and testing of the model using the selected variables. The selection of variables was accomplished using the LASSO method (Least Absolute Shrinkage and Selection Operator). The model performance was assessed using receiver operator characteristic curves (ROC) and the area under the curve (AUC).

**Results:**

Our analysis demonstrated a significant impact of clinical parameters (primary tumor site, presence of non‐bone metastases, steroids and opioid intake, food intake, and body mass index) and laboratory parameters (interleukin 8 [IL‐8], chloride levels, C‐reactive protein, white blood cell count, and lymphocyte count) on OS. Notably, different factors were associated with the different times for OS with only IL‐8 included both in the PMs for short‐ and long‐term OS. The AUC values for ROC curves for 3‐week, 24‐week, and 52‐week OS were 0.901, 0.767, and 0.806, respectively.

**Conclusions:**

We successfully developed three PMs for OS based on easily accessible clinical and laboratory parameters for patients referred to palliative RT for painful BMs. While our findings are promising, it is important to recognize that this was an exploratory trial. The implementation of these tools into clinical practice warrants further investigation and confirmation through subsequent studies with separate databases.

## 1 INTRODUCTION

Advanced cancer stage disease represents a unique clinical condition characterized by specific disease progression and features, rendering traditional prognostic prediction methods (staging) inadequate and unreliable. Physicians often face challenges in accurately estimating the prognosis of patients in this context. Christakis et al.^1^ found, in their prospective cohort study, that the estimated prognosis was 3–5 times longer than the actual survival time.

However, recent studies have improved the accuracy of prognosis prediction in patients with advanced‐stage cancers.^2^ Still, further developments of prognostic models to predict outcomes remains imperative in this clinical setting. Providing patients and their families with reliable prognostic information is crucial to address the psychological and social implications of end‐of‐life care. Moreover, the ability to anticipate the need for palliative and supportive therapies can help avoid unnecessary treatments. Specifically, the development of predictive models becomes particularly valuable in determining the suitability of referring patients with limited life expectancy for palliative radiotherapy (RT), as achieving maximum pain relief for certain symptoms may take up to 4 weeks.^3^ To this end, Mizumoto and colleagues devised a scoring system to predict survival in patients with spinal metastases, aiming to select the most appropriate RT treatment schedule and prevent overtreatment for patients with short life expectancy.^4^

To enable personalized treatment even for terminal cancer patients, numerous studies have explored the prognostic significance of various factors. In addition to traditional parameters related to tumor extent, other molecular, biological, and clinical factors, including treatment types and hospitalization, have shown potential prognostic implications.^5^ For instance, Maltoni et al.^6^ found a statistically significant correlation between survival and several parameters, such as clinical prediction of survival, performance status, cancer anorexia‐cachexia syndrome, dyspnea, and various biological factors (leukocytosis, lymphocytopenia, C‐reactive protein), in patients with advanced cancer.

However, the precision of individual parameters in outcome prediction is inevitably limited, as emphasized by the authors of that analysis. In contrast, the development of predictive models incorporating multiple parameters could offer more precise prognostication. The ProPART study^7^ assessed the possibility of identifying patients most suitable for palliative RT before treatment initiation (i.e., those at lower risk of temporary or definitive treatment interruptions). The analysis demonstrated that a prognostic tool based on multiple parameters (Palliative Prognostic [PaP] score) outperformed any single parameter in predictive power. Furthermore, several studies have shown that the clinical trajectories of patients with advanced cancer can be better predicted by different parameters or scores depending on the disease stage,^8^, ^9^ as confirmed by a recent systematic review of the European Society of Medical Oncology.^10^ In particular, the latter has identified three different categories within patients with advanced cancer: patients with a prognosis of at least a few months and receiving Disease Modifying Treatments (DMTs), subjects with a prognosis of weeks‐months no longer receiving DMTs, and patients with a prognosis of days not treated with DMTs.

Despite these advancements, there remains a clear imperative to develop increasingly accurate PMs in the context of terminal cancer. Consequently, the present study aims to optimize prognosis prediction in the setting of palliative RT for BMs by utilizing a large multicenter case series.

## 2 MATERIALS AND METHODS

### 2.1 Study design

This study constitutes a sub‐analysis of the PRAIS (Palliative Radiotherapy And Inflammation study) trial, a longitudinal observational study conducted across multiple European centers (Trondheim, Ålesund, Oslo in Norway; Milan and Meldola in Italy; Lleida in Spain; Hull in the United Kingdom) to collect data from patients referred for palliative RT due to cancer‐induced bone pain.^11^ For our analysis, a total of 567 patients in the PRAIS trial database were included. The primary objective was to assess the correlation between clinical and laboratory parameters with the probability of survival at three distinct time points (short: 3 weeks; intermediate: 24 weeks; prolonged: 52 weeks) and potentially develop predictive models for prognosis in this context.

### 2.2 End points

The selected endpoints for this analysis were overall survival at 3, 24, and 52 weeks after the start of RT. These three time points were arbitrarily chosen as indicative of short‐, intermediate‐, and long‐term prognosis, respectively.

### 2.3 Inclusion criteria

All patients enrolled in the PRAIS study were considered eligible for our analysis. The trial included patients with verified cancer diagnosis, radiologically confirmed BMs, who were scheduled to undergo palliative RT for painful BMs.

### 2.4 Collected data and follow‐up

The PRAIS trial collected patient assessments at baseline and at various time points after RT. Each evaluation comprised two case report forms (CRFs) completed by the patient and physician, respectively. The CRFs included information on demographic data, clinical variables, Quality of Life evaluation (EORTC QLQ‐C15 PAL), nutritional status assessment (PG‐SGA), depression levels (PHQ9), and pain assessment (LANSS). Additionally, blood samples for standard clinical chemistry and serum biomarker analysis were collected both at baseline and during all follow‐up visits.^12^ A comprehensive list of outcomes and characteristics, including patient and tumor‐related data, treatment details, and laboratory parameters, is provided in Data S1, respectively.

### 2.5 Treatment

The study included patients with solid tumors referred to palliative RT centers. Previous, concomitant, and subsequent treatments with RT (even in the same anatomical site), chemotherapy, hormone therapy, targeted therapies, and immunotherapy were permitted. Furthermore, prior, concomitant, and subsequent use of any palliative care, such as analgesics, steroids, antidepressants, antiemetics, sedatives, laxatives, prokinetics, and other drugs, were eligible for inclusion in the study.

### 2.6 Variables selection and machine learning modeling

The LASSO method (Least Absolute Shrinkage and Selection Operator) and Machine learning techniques were employed in the following steps: (i) selection of reliable prognostic variables, and (ii) training and validation of the model using the subset of selected variables. LASSO is a powerful supervised algorithm that enables the identification of variables influencing the “death” event and determining which ones to include or eliminate from the model. With the final significant covariates, classification and regression tree analysis (CART) machine learning models were created to distinguish between deceased and non‐deceased patients. CART, a decision tree‐based data mining tool, can automatically search for patterns and identify data links, even in extensive datasets. The CART model is represented as a binary tree, with each root node representing an input feature and a split point on that feature. The leaf nodes in the tree contain an output variable used for forecasting.

All models were cross‐validated using a 5‐fold cross‐validation repeated 100 times. Model performance was assessed using receiver operator characteristic curves (ROC) and the area under the curve (AUC).

### 2.7 Statistical analysis

Descriptive statistics were used to summarize continuous variables (number of cases, mean, standard deviation, median, minimum, and maximum), and categorical variables were presented as counts of patients and percentages. Overall survival was defined as the time from enrollment in the study to the date of death from any cause or the date of the last available information. Survival curves were estimated using the Kaplan–Meier product‐limit method and compared using the log‐rank test.

Categorical data were transformed into numeric data to satisfy preprocessing criteria for machine learning. One‐hot encoding converted each level of each categorical feature into a new binary feature.

The effectiveness of each model was evaluated in terms of discrimination and calibration. Discrimination, that is, the ability of a model to stratify between high and low risk of the predicted outcome, was quantified with the area under the receiving operating characteristic curve (AUC). The calibration of the ML models was evaluated to assess the consistency between predicted and actual probability of the outcome. Results are displayed as calibration plots, showing the relationship between the observed outcome frequencies and the predicted probabilities for groups of patients. Also, the Brier scores, that is, the squared differences between predicted and observed outcomes were estimated for each model.

Statistical analyses were performed using the XLSTAT (Addinsoft, New York, USA) and Python 3.8 (Python Software Foundation, OR, USA) statistical packages.

### 2.8 Ethical issues

The study received approval from the Area Vasta Romagna Ethics Committee (code: L2P1517; May 17, 2017) and was conducted in accordance with the 1964 Helsinki Declaration and its later amendments, as well as Good Clinical Practice guidelines. Written informed consent was obtained from all individual participants included in the study. The manuscript does not include any identifiable human data.

## 3 RESULTS

### 3.1 Patients and treatment characteristics

Table 1 displays the characteristics of the 567 patients included in the study. The average age was 66 (±10.7) years, with a higher representation of male individuals (61.4%). Most patients were of Caucasian ethnicity, and the primary tumor sites were primarily prostate (25.6%), breast (19.6%), and lung (17.8%). Among the patients, 57.9% had a Karnofsky Performance Status (KPS) score ≥80, and 70% were managed as outpatients. The median overall survival was 8.1 months (95% CI: 7.1–9.0). BMs were most commonly located in the spine (46%), and pelvis (33%). Approximately 37% of the patients received a single fraction of RT, and 80% and 33% of patients used opioids and steroids, respectively (Table 2). Despite these treatments, 60% of patients experienced poorly controlled pain at either the 3‐ or 8‐week follow‐up.

**TABLE 1:** Patients characteristics.

	No. (%)
Age (years): median (range, IQR)	67 (29–91, 59–74)
Gender	
Male	348 (61.4)
Female	219 (38.6)
Weight (kg): median (range, IQR)	76 (40–180, 66–87)
BMI	
<18.5	20 (3.8)
18.5–24.9	237 (45.0)
25.0–29.9	186 (36.3)
≥30.0	84 (15.9)
Unknown/missing	40
Ethnicity	
Caucasian	563 (99.3)
African	0
Asian	3 (0.5)
Other	1 (0.2)
Charlson's comorbidity index: median (range, IQR)	0 (0–6, 0–1)
0	366 (64.6)
1	119 (21.0)
2	47 (8.3)
3	20 (3.5)
4	9 (1.6)
5	2 (0.3)
6	4 (0.7)

Abbreviations: BMI, body mass index; IQR, interquartile range.

**TABLE 2:** Treatment characteristics.

	No. (%)
Radiotherapy	
Number of planned fractions	
1	209 (36.9)
2	2 (0.3)
3	2 (0.3)
4	9 (1.6)
5	156 (27.5)
6	2 (0.3)
7	12 (2.1)
8	2 (0.3)
9	1 (0.2)
10	152 (26.8)
12	7 (1.2)
13	8 (1.4)
15	1 (0.2)
20	2 (0.3)
25	2 (0.3)
Dose per fraction (Gy)	
1	1 (0.2)
2	8 (1.4)
3	174 (30.7)
4	165 (29.1)
5	7 (1.2)
6	1 (0.2)
8	211 (37.2)
Previous irradiation in the same anatomical site	
No	507 (92.0)
Yes	44 (8.0)
Missing	16
Chemotherapy	
Previous	307
Concurrent	136
Radiotherapy	
Previous (same or other sites)	276
Concurrent (other sites)	24
Hormonal therapy	
Previous	129
Concurrent	184
Opioids	
No	111 (19.6)
Yes	455 (80.4)
Steroids	
No	376 (66.7)
Yes	188 (33.3)

### 3.2 Variables selection and machine learning modeling

**FIGURE 1:**
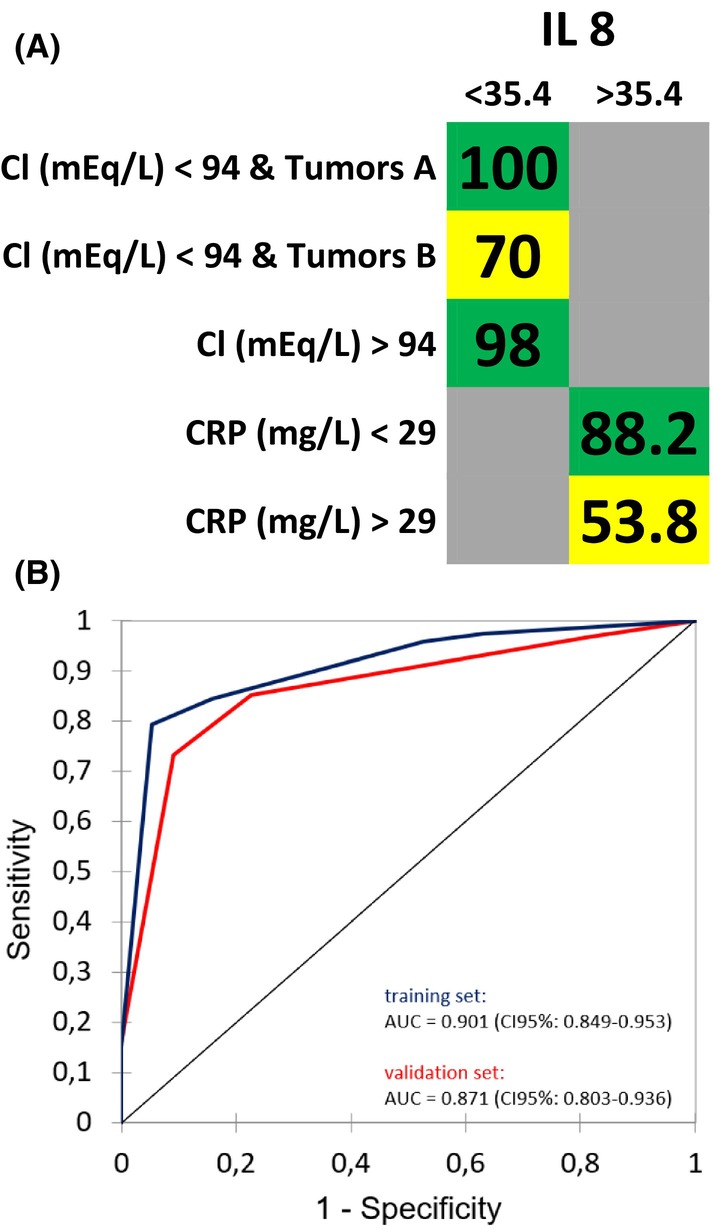
(A) Overall Survival probability within 3 weeks; the numbers in the cells are percentages; green background: Low risk; yellow background: Intermediate risk; in cells with a gray background, the parameter indicated in the first column on the right should not be considered; Chloride (Cl) is measured in milliequivalent per liter; C‐reactive protein (CRP) is measured in milligrams per liter; Interleukin‐8 (IL8) is measured in pictograms per milliliter. Tumors A: Primary cancer is breast or prostate or lung. Tumors B: Other primary cancers. (B) ROC curves of the predictive model for 3 weeks survival for the training (blue) and validation (red) set.

**FIGURE 2:**
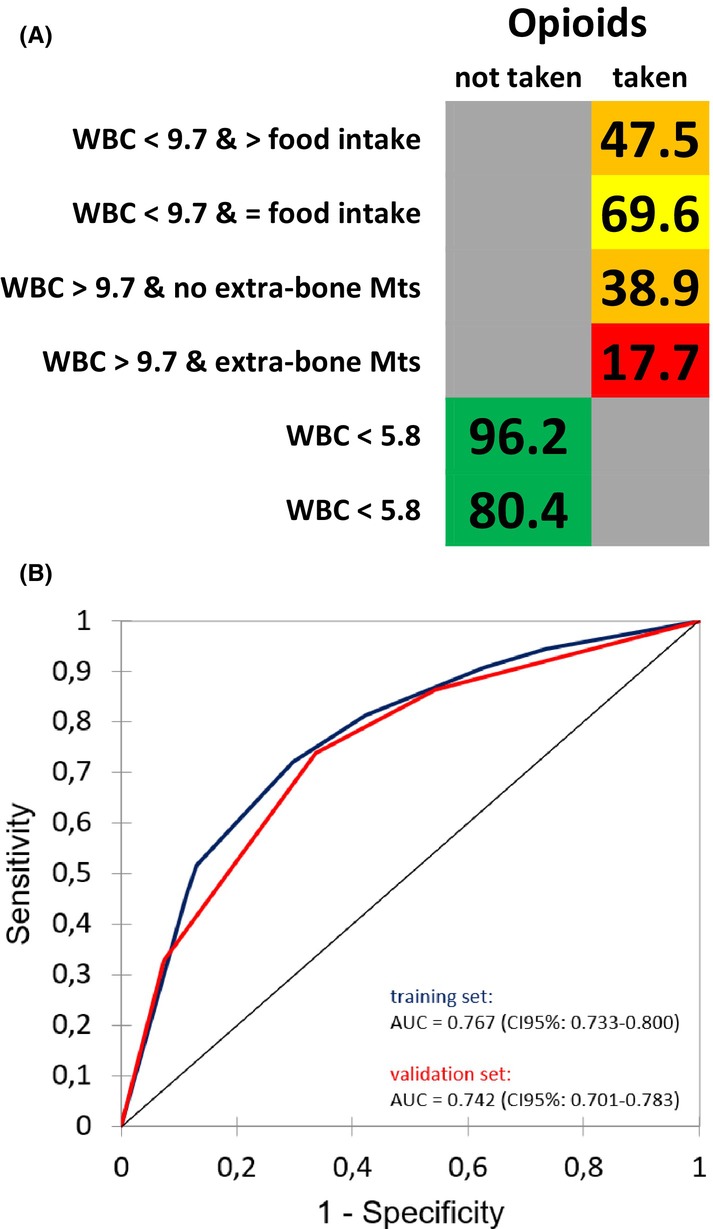
(A) Overall Survival probability within 24 weeks; the numbers in the cells are percentages; green background: Low risk; yellow background: Intermediate risk; orange background: High risk; red background: Very high risk; in cells with a gray background, the parameter indicated in the first column on the right should not be considered; > food intake, Increased food intake compared to the previous month; = food intake, Food intake similar to the previous month; Mts: Metastases; WBC, White Blood Cells (10^3^/μl). (B) ROC curve of the predictive model for 24 weeks survival for the training (blue) and validation (red) set.

**FIGURE 3:**
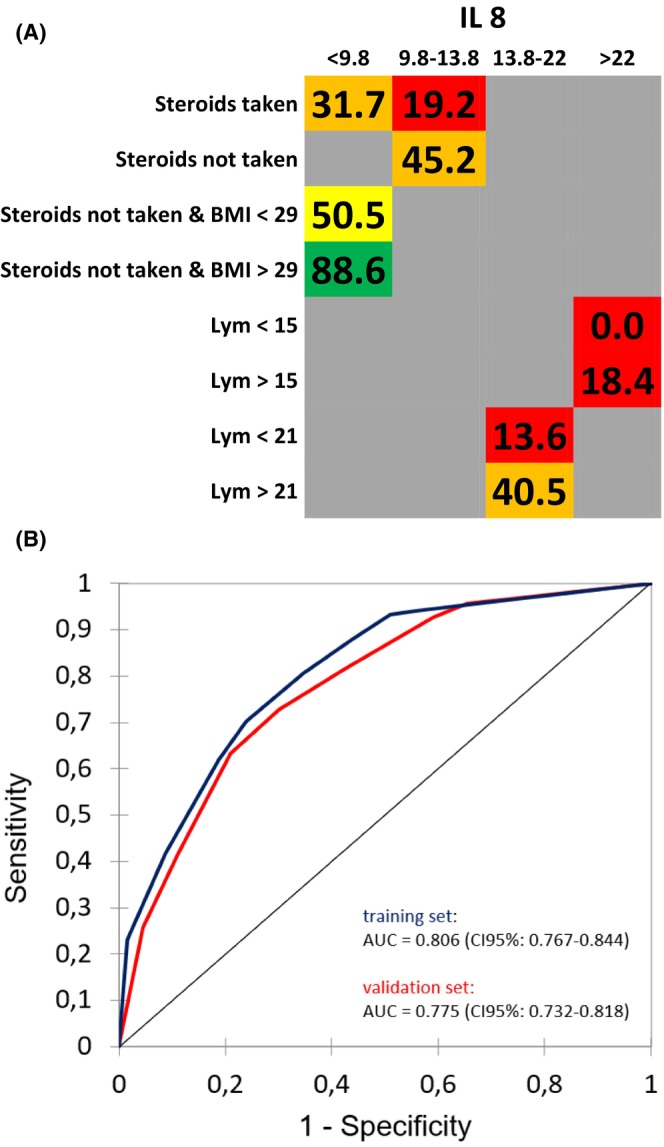
(A) Overall Survival probability within 52 weeks; the numbers in the cells are percentages; green background: Low risk; yellow background: Intermediate risk; in cells with a gray background, the parameter indicated in the first column on the right should not be considered; BMI, Body mass index; Lymphocytes (Lym) expressed as (absolute lymphocyte count/total white blood cell count) ×100. (B) ROC curve of the predictive model for 52 weeks survival for the training (blue) and validation (red) set.

The prediction of three‐week survival was dependent on four parameters: Interleukin 8 (IL‐8) concentration, serum chloride concentration, site of the primary tumor, and blood C‐reactive protein levels (CRP). In the training and validation cohort, the corresponding AUC of the ROC curves for this model were 0.901 (CI 95%: 0.849–0.953) and 0.871 (CI 95%: 0.803–0.936), respectively, indicating a high level of accuracy in predicting short‐term survival. For the prediction of 24‐week survival, four parameters were identified: opioid intake, white blood cell (WBC) count, food intake characteristics, and the presence of extra‐bone metastases. In the training and validation cohort, the corresponding AUC were 0.767 (CI 95%: 0.733–0.800) and 0.742 (CI 95%: 0.701–0.783), respectively, suggesting a moderate predictive performance for this model. Additionally, for the prediction of 52‐week survival, four parameters were found to be significant: IL‐8 levels, steroid intake, BMI, and lymphocyte count. In the training and validation cohort, the corresponding AUC were 0.806 (CI 95%: 0.767–0.844) and 0.775 (CI 95%: 0.732–0.818), respectively, indicating a good predictive performance for this model. Figures 1, 2, 3 display the type of impact (negative or positive) and the cut‐offs defined by the model for all parameters, along with the corresponding ROC curves, further illustrating the predictive performance of each model.

**FIGURE 4:**
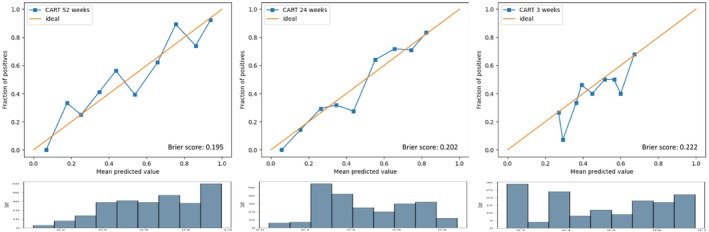
Calibration curves for the three machine learning models.

The calibration curves and the Brier scores for the three models are presented in Figure 4, demonstrating the consistency between the predicted values and the real outcome. The Brier scores range from 0.195 to 0.222.

## 4 DISCUSSION

Our study, derived from the database of the multicenter PRAIS trial, presents a heterogeneous patient population encompassing various metastatic cancers with different degrees of tumor spread. The primary objective of this study was to determine whether the analysis of data from a large cohort of patients, encompassing clinical, tumor‐related, treatment, and laboratory parameters, could aid in the development of predictive models for overall survival using artificial intelligence‐based methods.

To the best of our knowledge, this study represents the most comprehensive analysis to date. It encompasses a total of 49 laboratory parameters, along with 10 patient‐related, 8 tumor‐related, and 7 treatment‐related parameters. The inclusion of such an extensive range of variables provides a more comprehensive and in‐depth understanding of the factors influencing overall survival in the setting of palliative RT.

The analysis revealed the impact of clinical parameters (primary tumor site, presence of non‐bone metastases, steroid and opioid use, food intake, and BMI) and laboratory parameters (IL‐8, chloride, CRP, WBC, and lymphocytes) on overall survival. Interestingly, some traditionally considered prognostic factors, such as histological type and performance status, were excluded from the models. It is important to note that the exclusion of these variables was not a subjective decision on our part but rather a result of the data‐driven nature of the machine learning algorithm used in our analysis. The LASSO method, which we employed for variable selection, penalizes the inclusion of less predictive variables. In this case, the algorithm identified that these clinical variables did not significantly contribute to the predictive power of the models for overall survival within our specific dataset. This does not necessarily negate the prognostic impact of these factors in other contexts or datasets, but highlights that within our cohort, other variables provided stronger predictive value. Notably, only IL‐8 emerged as a predictor for short‐ and long‐term overall survival, while the remaining parameters were included in only one of the three predictive models.

IL‐8 is a pro‐inflammatory factor belonging to the CXC chemokine family. Initially named neutrophil‐activating peptide‐1 due to its potent chemotactic activity on granulocytes in inflammatory and immune diseases,^13^, ^14^ recent research has highlighted its critical role in cancer invasion, angiogenesis, and metastasis.^15^, ^16^, ^17^, ^18^ IL‐8 has also emerged as a significant component of the tumor microenvironment, often referred to as the “soil” of cancer cells, with cancer‐stroma interactions becoming critical determinants of cancer behavior.^19^, ^20^

In various cancers, stromal cells can produce IL‐8, influencing the invasion and metastasis potential of cancer cells. Additionally, cancer cells themselves can secrete IL‐8 in an autocrine or paracrine manner, as seen in breast cancer,^21^ gastric cancer,^16^ colon cancer,^22^ cervical cancer,^23^ pancreatic cancer,^20^, ^24^ and leukemia.^25^, ^26^

Multiple studies have shown correlations between IL‐8 levels and treatment outcomes in cancer patients. A recent review^27^ highlighted the significant association between high IL‐8 levels and shorter overall survival and progression‐free survival in colorectal cancer patients. In advanced melanoma and non‐small cell lung cancer (NSCLC) patients treated with anti‐PD‐1 mAbs, changes in serum IL‐8 levels were studied. Responding patients showed significant decreases in serum IL‐8 levels between baseline and best response and significant increases upon progression. Early decreases in IL‐8 levels were linked to longer overall survival, suggesting IL‐8 could predict clinical benefit from immune checkpoint blockade in melanoma and NSCLC patients.^28^

IL‐8 prognostic value was further assessed in pancreatic cancer, where it was found to be overexpressed in pancreatic adenocarcinoma samples compared to matched para‐cancer tissues or chronic pancreatitis.^29^ In hepatocellular carcinoma, high IL‐8 expression was significantly and independently associated with poor overall survival and disease‐free survival.^30^ Additionally, in gastric cancer, high pre‐therapeutically serum IL‐8 levels were associated with poor responses to platinum‐based therapy.^31^ These findings collectively emphasize the significance of IL‐8 in cancer progression and suggest its potential as a valuable prognostic marker and therapeutic target in various cancer types, as confirmed in our analysis.

A noteworthy aspect of our analysis is the observed adverse impact on prognosis associated with the use of opioid and steroid medications. The interpretation of this finding and its implications for clinical management are multifaceted. Several studies have suggested potential immunosuppressive effects of these treatments, particularly relevant in patients undergoing immunotherapy.^32^, ^33^ These findings underscore the importance of a judicious use of these medications, emphasizing minimal effective doses and appropriate treatment durations aligned with clinical objectives.^34^, ^35^, ^36^ It is crucial to note that the negative effect of these medications has not achieved unanimous consensus; conversely, some evidence suggests that this correlation could be a mere association with worse prognosis among patients necessitating more aggressive supportive therapies.^37^ Lastly, it is plausible that patients requiring these treatments might inherently face a worse prognosis if deprived of them.^6^, ^38^, ^39^, ^40^, ^41^, ^42^

An unexpected finding was that low serum chloride concertation was associated with a short‐term poor prognosis. This may reflect that inflammation and advanced disease cause fluid accumulation of free water which may be observed as low chloride and sodium concentrations. Similarly, organ failure could cause accumulation of other negatively charged molecules causing a compensatory lower chloride concentration in order to maintain isoelectric equilibrium. Furthermore, chloride serum concentration is a potential proxy for general inflammation and organ failure, and this explanation is supported by the impact of CRP in the same short‐term model.

Several predictive models have been proposed in the setting of palliative RT, including the NRF risk factor score, the Katagiri score, the Mizumoto score for spinal metastases, the TEACHH score, and the PaP score. Our results contribute to the growing body of literature using artificial intelligence‐based tools to improve prognostication in this context.^4^, ^7^, ^43^, ^44^, ^45^

Other recent studies have evaluated the possibility of predicting the prognosis of cancer patients with BMs using machine learning systems (Table 3). These studies represent recent efforts to employ machine learning systems to predict the prognosis of cancer patients with BMs. Each study utilized different machine learning techniques and prediction parameters, including clinical characteristics, treatment modalities, and other relevant factors. The findings from these studies indicate the potential of machine learning‐based predictive models to improve prognostication in this clinical setting, offering more accurate and personalized treatment decisions.^46^, ^47^, ^48^, ^49^, ^50^, ^51^ However, further validation and generalizability are essential to ensure the clinical utility of these models.

**TABLE 3:** Recent studies on prognosis prediction in cancer patients with bone metastases using machine learning systems.

Authors (year)	Methods/Techniques	Prediction parameters	Findings
Huang et al.^46^	Extreme Gradient Boosting (XGBoost)	Tumor size, age, race, sex, primary site, histological subtype, grade, laterality, T stage, N stage, surgery, RT, chemotherapy, distant metastases (lung, brain, and liver), marital status	Developed an XGBoost‐based model with high accuracy for predicting the 1‐year survival rate of NSCLC patients with BM. XGBoost showed superior performance compared to other models. The model can assist clinicians in designing more rational and effective therapeutic strategies.
Elledge et al.^47^	Bone Metastases Ensemble Trees	Symptomatic BMSBM patients treated with palliative RT	Validated the Bone Metastases Ensemble Trees for Survival (BMETS) ML model on external data sets, demonstrating its validity, stability, and feasibility of dynamic modeling. BMETS accurately estimated survival time following palliative RT for symptomatic BM.
Cui et al.^48^	Logistic Regression, XGBoosting, Random Forest, Neural Network, Gradient Boosting Machine, Decision Tree	Age, primary site, histology, race, sex, T stage, N stage, brain metastasis, liver metastasis, lung metastasis, cancer‐directed surgery, RT, chemotherapy	Developed several prediction models using ML to predict 3‐month mortality among lung cancer patients with BM based on easily available clinical data. The gradient boosting machine approach showed the best performance in prediction accuracy. High‐risk patients identified by the model may benefit from tailored treatment strategies.
Le et al.^49^	Extreme Gradient Boosting (XGB), Logistic Regression, Random Forest, Naive Bayes	Age, marital status, grade, T stage, N stage, tumor size, brain metastasis, liver metastasis, lung metastasis, surgery	Developed and validated predictive models using ML algorithms for overall survival of clear cell renal cell carcinoma (ccRCC) patients with BM. All four ML algorithms showed good performance in predicting 1‐year and 3‐year survival rates for ccRCC‐BM patients. These models can serve as valuable tools in clinical decision‐making.
Li et al.^50^	COX Regression, XGBoost	Various clinical‐pathological characteristics	Developed an XGBoost ensemble ML model to predict the prognosis of breast cancer patients with initial BM. The model demonstrated high sensitivity, specificity, and accuracy, providing a valuable tool for predicting survival outcomes and guiding treatment decisions in this patient population.
Long et al.^51^	Ensemble ML	Age, marital status, tumor stage, node stage, fibrosis score, AFP level, tumor size, lung metastases, cancer‐directed surgery, RT, chemotherapy	Developed and validated an ensemble ML model for predicting early mortality among patients with BM of hepatocellular carcinoma. The model demonstrated promising prediction performance for early mortality in hepatocellular carcinoma patients with BM and can aid in clinical decision‐making.

Abbreviations: BM, bone metastases; ML, machine learning; RT, radiation therapy.

We recognize that our study has limitations. The patient population was predominantly Caucasian from European centers, potentially limiting the generalizability of the results to other geographical and ethnic contexts. Additionally, the prediction models for short‐ and long‐term overall survival require IL‐8 levels, which may necessitate additional blood samples beyond routine clinical practice. External validation of our model on an independent patient cohort is also lacking, warranting further collaboration with other centers for validation.

The findings of our and other studies^52^, ^53^, ^54^ emphasize the need for continued research to develop predictive models in patients with BMs, considering their value in personalizing treatment decisions. The ideal scenario involves creating readily available and user‐friendly online tools to facilitate the incorporation of predictive models into routine clinical practice.

## 5 CONCLUSION

Our study demonstrated that with minimal data, primarily derived from simple patient questioning and routine blood tests, it is possible to predict short, medium, and long‐term overall survival with promising accuracy. Interestingly, the best performance was observed in the 3‐week predictive model of overall survival, with an impressive area under the curve (AUC) value of 0.901. This result is noteworthy, as the model enables the identification of patients with a high risk (53%) of three‐week mortality, indicated by elevated levels of IL‐8 (>35.4) and CRP (>29). For these patients, palliative RT could be reasonably considered only in cases of severe pain that is not pharmacologically treatable, thereby providing valuable insights for clinical decision‐making. However, it is important to note that this was an exploratory trial, and these findings need to be confirmed with separate databases to ensure their robustness and generalizability.

## AUTHOR CONTRIBUTIONS

**Romina Rossi:** Conceptualization (equal); formal analysis (equal); validation (equal). **Federica Medici:** Conceptualization (equal); formal analysis (equal); project administration (equal); writing – original draft (equal). **Ragnhild Habberstad:** Conceptualization (equal); formal analysis (equal). **Pal Klepstad:** Conceptualization (equal); data curation (equal). **Savino Cilla:** Formal analysis (equal). **Monia Dall'Agata:** Data curation (equal). **Stein Kaasa:** Data curation (equal). **Augusto Tommaso Caraceni:** Data curation (equal); formal analysis (equal). **Alessio Giuseppe Morganti:** Conceptualization (equal); formal analysis (equal); writing – review and editing (equal). **Marco Maltoni:** Conceptualization (equal); formal analysis (equal); writing – review and editing (equal).

## FUNDING INFORMATION

This research received no external funding.

## CONFLICT OF INTEREST STATEMENT

The authors declare that the research was conducted in the absence of any commercial or financial relationships that could be construed as a potential conflict of interest.

## ETHICS STATEMENT

The study was conducted according to the guidelines of the Declaration of Helsinki and approved by the Area Vasta Romagna Ethics Committee (code: L2P1517; May 17, 2017).

## INFORMED CONSENT STATEMENT

Informed consent was obtained from all subjects involved in the study. This secondary analysis hasn't been previously submitted to any other journal.

## Supporting information


Data S1.

## Data Availability

The data that supports the findings of this study are available in the supplementary material of this article.
