# Supplementary material for: Development of a predictive model for patients with bone metastases referred to palliative radiotherapy: Secondary analysis of a multicenter study (the PRAIS trial)
Source: Cancer Med. 2024 Oct 10;13(19):e70050. doi: 10.1002/cam4.70050 (PMC11467037; doi:10.1002/cam4.70050)
Supplement: Supplementary file 1 — Data S1. [file CAM4-13-e70050-s001.docx]

**SUPPLEMENTARY MATERIALS**

**1. List of outcomes included in the PRAIS study (in bold are shown the outcomes considered in this analysis)**

**Death within 3 weeks**

Death within 8 Weeks

**Death within 24 Weeks**

**Death within 52 Weeks**

New Metastases within 3 Weeks

New Metastases within 24 Weeks

New Metastases within 52 Weeks

Systemic Therapy at 24 Weeks

Systemic Therapy at 52 Weeks

Radiotherapy at 24 Weeks

Radiotherapy at 52 Weeks

**2. List of patient’s characteristics considered in this analysis**

1. Gender
2. Weight (Kg)
3. Height (M)
4. BMI
5. Karnofsky Performance Status
6. Department Site
7. Medium NRS in the last 24 hours
8. Worst NRS in the last 24 hours
9. Weight of the Last 2 Weeks
10. Food Intake in the last Month

**3. List of tumor characteristics considered in this analysis**

1. Primary tumor
2. Liver Metastases
3. Central Nervous System Metastases
4. Lung Metastases
5. Other Metastases
6. Extension of bone metastases to soft tissues
7. Osteolitic bone metastases
8. Metastases of the spine (rachis)

**4. List of treatment characteristics considered in this analysis**

1. Site of radiotherapy treatment
2. Extension of bone metastases to soft tissues
3. Osteolitic bone metastases
4. Metastases of the spine (rachis)
5. Number of radiotherapy fractions delivered
6. Dose administered for fraction
7. Reirradiation

**5. List of laboratory parameters considered in this analysis**

1. HEMOGLOBIN
2. WHITE BLOOD CELLS
3. LYMPHOCITES
4. NEUTROPHILS
5. BANDS
6. BASOPHILS
7. EOSINOPFILS
8. MONOCITES
9. PLATELETS
10. C REACTIVE PROTEIN
11. CREATININE
12. UREA
13. ALBUMIN
14. BILIRUBINE
15. POTASSIUM
16. SODIUM
17. CHLORIDE
18. CALCIUM
19. PHOSPHORUS
20. MAGNESIUM
21. TRIGLYCERIDES
22. VITD
23. TNF A
24. IFNG
25. IL1B
26. IL1RA
27. -IL2
28. IL4
29. IL5
30. IL6
31. IL7
32. IL8
33. IL9
34. IL10
35. IL12P70
36. IL13
37. IL15
38. IL17A
39. MCP1MCAF
40. IP10
41. EOTAXIN
42. MIP1A
43. MIP1B
44. RANTES
45. GCSF
46. GMCSF
47. BASICFGF
48. PDGFBB
49. VEGF
